# Multiomics surface receptor profiling of the NCI-60 tumor cell panel uncovers novel theranostics for cancer immunotherapy

**DOI:** 10.1186/s12935-022-02710-y

**Published:** 2022-10-11

**Authors:** Simon Heumos, Sandra Dehn, Konstantin Bräutigam, Marius C. Codrea, Christian M. Schürch, Ulrich M. Lauer, Sven Nahnsen, Michael Schindler

**Affiliations:** 1 Quantitative Biology Center (QBiC), University of Tübingen, 72076 Tübingen, Germany; 2 Biomedical Data Science, Dept. of Computer Science, University of Tübingen, 72076 Tübingen, Germany; 3 Institute for Medical Virology and Epidemiology of Viral Diseases, University Hospital Tübingen, Tübingen, Germany; 4 Institute of Pathology, University of Bern, 3008 Bern, Switzerland; 5 Department of Pathology and Neuropathology, University Hospital and Comprehensive Cancer Center Tübingen, Tübingen, Germany; 6 Department of Internal Medicine VIII, Medical Oncology and Pneumology, Virotherapy Center Tübingen (VCT), Medical University Hospital Tübingen, 72076 Tübingen, Germany; 7 German Cancer Consortium (DKTK), German Cancer Research Center (DKFZ), Partner Site Tübingen, 72076 Tübingen, Germany

**Keywords:** Immunotherapy, cancer, Multiomics, Theranostics, Flow cytometry, FACS, NCI-60, Receptorome

## Abstract

**Background:**

Immunotherapy with immune checkpoint inhibitors (ICI) has revolutionized cancer therapy. However, therapeutic targeting of inhibitory T cell receptors such as PD-1 not only initiates a broad immune response against tumors, but also causes severe adverse effects. An ideal future stratified immunotherapy would interfere with cancer-specific cell surface receptors only.

**Methods:**

To identify such candidates, we profiled the surface receptors of the NCI-60 tumor cell panel via flow cytometry. The resulting surface receptor expression data were integrated into proteomic and transcriptomic NCI-60 datasets applying a sophisticated multiomics multiple co-inertia analysis (MCIA). This allowed us to identify surface profiles for skin, brain, colon, kidney, and bone marrow derived cell lines and cancer entity-specific cell surface receptor biomarkers for colon and renal cancer.

**Results:**

For colon cancer, identified biomarkers are CD15, CD104, CD324, CD326, CD49f, and for renal cancer, CD24, CD26, CD106 (VCAM1), EGFR, SSEA-3 (B3GALT5), SSEA-4 (TMCC1), TIM1 (HAVCR1), and TRA-1-60R (PODXL). Further data mining revealed that CD106 (VCAM1) in particular is a promising novel immunotherapeutic target for the treatment of renal cancer.

**Conclusion:**

Altogether, our innovative multiomics analysis of the NCI-60 panel represents a highly valuable resource for uncovering surface receptors that could be further exploited for diagnostic and therapeutic purposes in the context of cancer immunotherapy.

**Supplementary Information:**

The online version contains supplementary material available at 10.1186/s12935-022-02710-y.

## Background

Implementation of cancer immunotherapy by immune checkpoint inhibitors (ICIs) is one of the most recent transforming developments in oncology that strongly helped to improve overall survival of patients suffering from various cancers [1]. Its principle is based on the antibody-mediated blockage of inhibitory immune signaling exerted by tumor cells to unleash the immune system, with the overall goal to achieve a sustainable tumor elimination by the host’s intrinsic immune response. The first established ICIs target the PD1-PDL1 inhibitory axis on T cells to activate the cytotoxic T lymphocyte (CTL) response [1] and block signaling of the T cell inhibitory receptor CTLA-4 [2]. In this context, alternative strategies are to activate not only T cells but also NK-cells [3], or to target general immune-inhibitory signaling axes, i.e., the CD47-SIRPα pathway [4]. Conceivably, due to the nature of this approach, cancer immunotherapy via ICIs is unspecific, can have severe adverse effects and has a certain risk of complete therapy failure or could even result in an aggravation of the addressed malignancies [5]. Hence, there are ongoing efforts to improve immunotherapies, especially in terms of specificity, so that only or at least mainly tumor cells are killed [6].

Such approaches are based, for example, on bispecific antibodies that bind to a tumor antigen and are designed to crosslink T cells to the tumor cells via secondary binding to CD3 or another T cell-specific antigen [7]. In this way, cytotoxic T cells should be specifically recruited to and kill malignant tumor cells. This concept is further elaborated to recruit NK cells, and also has been developed in the context of chimeric antigen receptor (CAR) T cells [7, 8]. CAR T cells are engineered to express an artificial T cell receptor tumor-specific antigen. Altogether, efforts are undertaken to improve efficacy of immunotherapy and reduce side-effects by specific targeting of malignant tumor cells.

The key to improving and precisely targeting cancer immunotherapy is the knowledge of tumor-specific biomarkers accessible at the cell surface. Therefore, we hypothesized that a novel systematic screening of cancer cell lines using a distinct comprehensive flow cytometric approach should enable the identification of hitherto unidentified cancer entity-specific cell surface receptors. For this, we took advantage of the NCI-60 tumor cell panel, a collection of 60 different human cancer cell lines that were established to facilitate systematic screening of anti-tumor drugs (https://dtp.cancer.gov/discovery_development/nci-60/cell_list.htm) [9–12].

We systematically characterized the expression of 332 receptors on the surface of the NCI-60 tumor cell collection using an array of flow cytometry-applicable antibodies. The NCI-60 panel has already been comprehensively characterized via transcriptomics and proteomics [13, 14]. While these latter approaches facilitate the identification of tumor biomarkers, they do not give any information on differential cell surface expression which is a prerequisite to exploit them as immunotherapeutic targets. Therefore, building on these high-quality public data sources, we analyzed the receptorome using flow cytometry and present an integrated three-layer multiomics approach. As a result of our cell surface receptor profiling using the NCI-60 tumor cell panel, we identified tumor biomarkers and immunotherapeutic targets that are readily accessible on the surface of human cancer cells via well-characterized antibodies. We anticipate new avenues for the development of highly specific and targeted immunotherapeutic approaches using the presented data as a resource.

## Methods

### Cell culture

The NCI-60 tumor cell panel from the US National Cancer Institute was purchased from Charles River Laboratories (Charles River Laboratories Inc., New York, NY, USA). All cell lines were cultivated in RPMI-1640 medium supplemented with 10% fetal calf serum (FCS), 2 mM l-glutamine and 100 µg ml^−1^ penicillin–streptomycin. Cells were cultured at 37 °C in an atmosphere of 5% CO_2_.

### Flow cytometric cell surface receptor screening

Before NCI-60 cells were used for flow cytometric analyses they were cultured from nitrogen stocks and allowed to grow for at least 2 weeks (four to five passages at maximum). Cells were detached by Accutase treatment and stained with the LegendScreen Human PE kit (BioLegend) using 332 PE-conjugated antibodies essentially as described before [15, 16]. Cell surface expression of the 332 receptors was measured via flow cytometry using the MACSQuant VYB Analyzer (Miltenyi Biotec). Flow cytometry data was analyzed with the FlowLogic (Miltenyi-Inivai) software to obtain the mean fluorescence intensity (MFI) values of each analyzed receptor.

### General data curation and quality control

For data quality control, to compare the receptor MFIs across 2 weeks, and for the MCIA analysis, R version 3.3.2 was used. For all other analysis the R version was 4.1.3 [17]. All analysis scripts including input and output data can be found at https://github.com/qbicsoftware/QMSFC. The repository comes with a detailed README and Anaconda environments to ensure reproducibility of the results [18]. For all data sets, as BR.MDAMB468 is not present in the microarray data, we removed it from the data set. We also removed tumor cell line ME-LOXIMVI as it is lacking any melanin production and therefore it is most likely not a melanoma cell line [19]. We harmonized the cell names and annotated the cells to the respective tissue type [20, 21].

### Flow cytometry data curation

For quality control, isotype control samples were analyzed separately from the data related to specific cell surface staining. The 10 isotype controls were removed from the full FACS data and considered individually as visible in Additional file 1: Fig. S1, Additional file 2: Fig. S2, Additional file 3: Fig. S3, Additional file 4: Fig. S4, Additional file 5: Fig. S5, Additional file 6: Fig. S6. Furthermore, for a set of cell lines, a second independent legend screen was conducted 1 week after the first sampling to check for reproducibility of the procedure, this data is available in Additional file 7: Dataset S1. The 25 cell lines of which a 2nd week measurement was performed were independently analyzed from the original FACS data (MFI values) set. For each of the cell lines, a between paired samples correlation test using the R function correlation test with method Spearman was executed. Before testing, a Shapiro-Wilk test of normality ensured that none of the measurements follow a normal distribution. For each receptor of each cell line pair, the log2 fold change was calculated. Furthermore, a between paired samples correlation test of all week 1 MFI value versus all week 2 MFI values was conducted. Before testing, an Anderson-Darling test for normality was performed. For further data analyses week 1 measurements only were used to have consistent data for all cell lines. FACS data (MFI values) of the receptor expression of the tumor NCI-60 cell line panel was log transformed (base 10). For all other downstream analyses, the raw expression values were used that are summarized in the Additional file 8: Dataset S2.

**Fig. 1:**
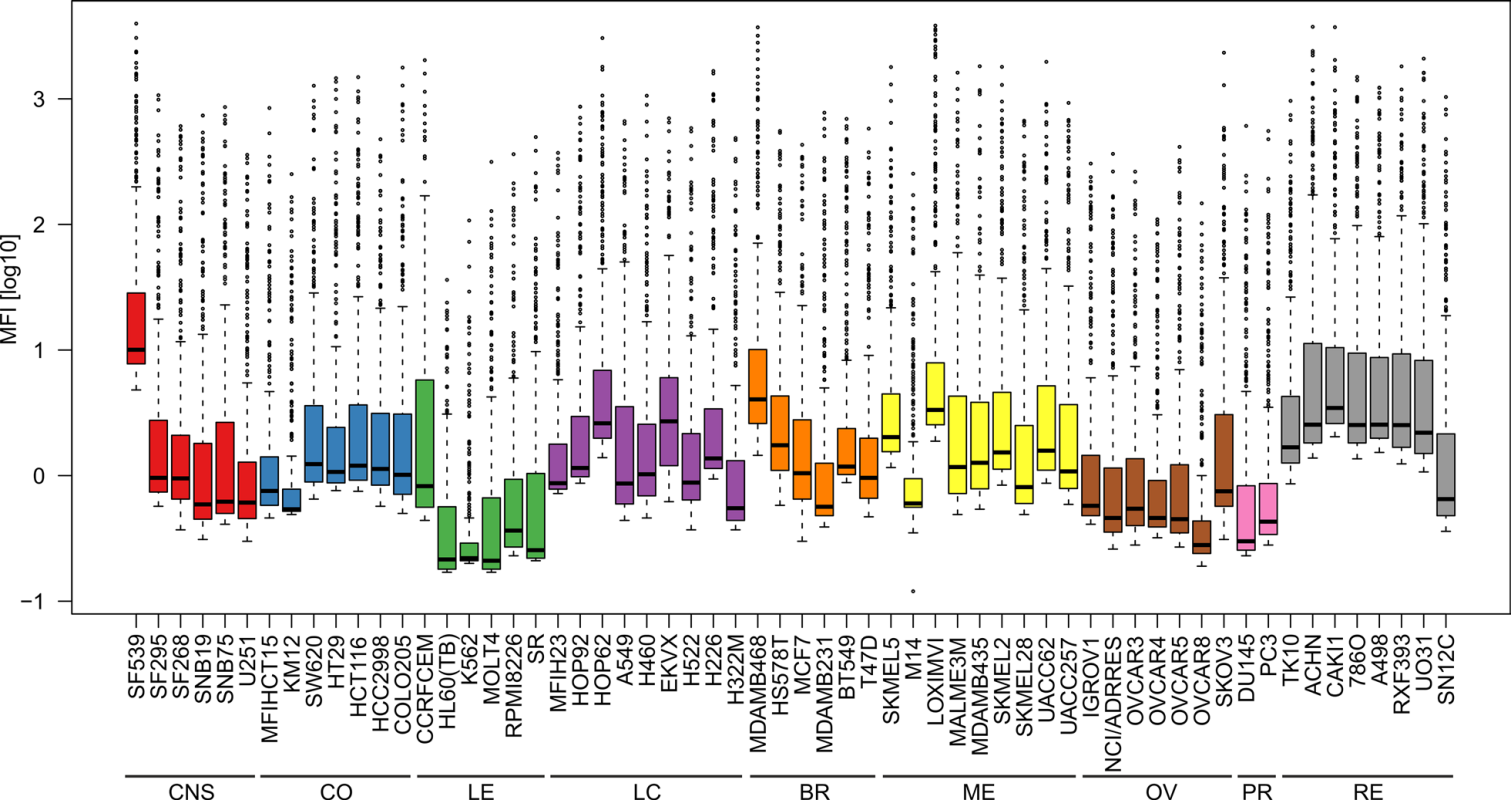
Flow cytometric profiling of the NCI-60 tumor cell panel. The NCI-60 tumor cell lines were analyzed for expression of 332 cell surface receptors by flow cytometry (see “Methods”). Shown is the distribution of the cell surface expression of all measured receptors as log_10_ mean fluorescence intensity (MFI). *CNS* cancer of the central nervous system, *CO* colon cancer, *LE* leukemia, *LC* lung cancer, *BR* breast cancer, *ME* melanoma, *OV* ovarian cancer, *PR* prostate cancer, *RE* renal cancer

### Microarray data curation

Robust multi-array average (RMA) [22] normalized microarray data of the NCI-60 cell lines was fetched from the Gene Expression Omnibus using accession number GSE32474 [12, 23–27]. The HGNC symbols for the Affymetrix U133 Plus 2.0 chip were downloaded from the Ensemble BioMart Portal [28]. The microarray probes were annotated with the HGNC symbols. All unannotated probes were discarded. We observed 37,989 distinct HGNC-Affymetrix identifiers and 19,473 unique HGNC symbols. We merged the expression values with the same HGNC symbol using their mean.

### Proteome data curation

MaxQuant [29] processed proteome data of the NCI-60 cell line panel was downloaded from PRIDE [30] using project number PXD005946 [14]. Label-free quantification (LFQ) values were log transformed (base 10), and previously identified contaminant proteins [14], labeled in the dataset with “CON” or “REV”, were removed. Both cell lines HOP92 and SR were two times in the proteomics data set. For each duplicate, we removed the cell line with more missing values. As the subsequent MCIA analysis can’t be performed with missing values, we only kept proteins with 60 measurements, 514 in total.

### Hierarchical clustering

Hierarchical clustering was performed using Spearman correlation [31] for the distance metric with ward.D2 for the agglomeration.

### MCIA

The omicade4 R package [19] was applied as an exploratory analysis to the transcriptomic study, the proteomic study and the FACS study of 58 cell lines. A customized version of the plotting function of omicade4 adds colors from the RColorBrewer package (https://CRAN.R-project.org/package=RColorBrewer). Using the information given in the sample and feature space of the MCIA, cell tissue type hits of FACS (LE, ME, CO, RE, CNS) were manually selected with selectVar. The resulting FACS receptors were annotated with gene identifiers for downstream comparison with the RNA-Seq recount2 data analysis.

### RNAseq data analysis

TCGA recount2 data of CNS, RE, CO, ME, LE was downloaded from the recount2 portal. For each of these, initial data processing was performed with the recount Bioconductor package [32]. Only samples with non-empty metadata were kept. Samples were filtered for “Primary Tumor” and “Solid Tissue Normal”. Only for the RE and CO tissue types there were “Solid Tissue Normal” samples. For CO, we obtained 500 tumor and 41 normal samples, and for RE we obtained 899 tumor and 129 normal samples, respectively. For both tissue type data, DESeq2 was applied in order to identify differentially expressed genes [33]. The experimental design formula was:

*~gdc_cases.demographic.gender + gdc_cases.demographic.race + gdc_cases.samples.sample_type*.

Genes were classified as differentially expressed with a p-adjusted value < 0.05.

### Human Protein Atlas data analysis

Available data for tissue protein expression in the Human Protein Atlas (HPA, [34]) was used for a systematic investigation of the eight differentially expressed molecules as identified by multi-omics. The expression of those eight molecules (CD24, CD26, CD106 [VCAM1], EGFR, SSEA4 [TMCC1], TIM1 [HAVCR1], SSEA3 [B3GALT5], TRA-1-60R [PODXL]) at the protein level was compared in normal renal tubules (in tissue cores of maximum 11 patients) vs. renal cell carcinoma, clear cell and non-clear cell type (in tissue cores of maximum 40 patients). For some of these proteins, immunohistochemistry data from multiple different antibody clones were available which is shown in Additional file 9: Table S1, all of which were included in the analysis. For each patient/kidney sample, up to two different tissue cores were available. Normal and tumor kidney samples were not matched. The cohort (n = 23) of renal cell carcinomas comprised 21 clear cell renal cell carcinomas (91.3%) and two non-clear cell renal cell carcinomas (8.7%) for CD106 (VCAM1). For EGFR (n = 40), 31 clear cell renal cell carcinomas (77.5%) and nine non-clear cell renal cell carcinomas (22.5%) were included.

Immunohistochemistry staining results were visually reviewed and jointly scored for each tissue core by two pathologists (K.B. and C.M.S.). The scoring was based on staining intensity and the amount of positive cells in a three-tiered manner, as follows: Intensity: 0: no expression; 1: weak expression; 2: moderate expression; 3: strong expression. Amount of positive cells: 0: none; 1: < 25%; 2: 25–75%; and 3: > 75% positive cells. Finally, the two values were multiplied, resulting in a modified immunoreactivity score (IRS, [35]) of values ranging between 0 and 9. In cases with two available tissue cores, the mean IRS was used for further analysis.

Data were visualized using GraphPad PRISM v.9.0.0. IRS scores of normal vs. tumor tissue were compared using the unpaired Mann-Whitney test. Two-tailed exact p-values < 0.05 were considered statistically significant. Limitations of the analyses: Firstly, the tissue cores of normal kidney tissue did not match tissue cores of renal adenocarcinoma. In addition, the sample size in the Human Protein Atlas was partially very limited and possibly affected statistical analysis. Moreover, some antibodies showed paradox and contradictory staining, an important technical limitation.

### The Cancer Proteome Atlas analysis

The Cancer Protein Atlas portal was accessed (https://tcpaportal.org/tcpa/differential_analysis.html) in order to explore the functional proteomics landscape of all renal cell cancer subtypes in regard to EGFR expression. On the web interface “By tumor type” and Pan-Can 32 were selected. For “Select tumor A” always “Kidney renal clear cell carcinoma (KIRC) (445 samples)” was chosen. For “Select tumor B” either “Kidney Chromophobe (KICH) (63 samples)” or “Kidney renal papillary cell carcinoma (KIRP) (208 samples)” was selected. The results were filtered by Protein Marker ID = EGFR and Gene(s) = EGFR.

## Results

### Cell surface receptor expression of the NCI-60 tumor cell panel

The NCI-60 tumor cell panel is a collection of 60 different human cancer cell lines representing 9 different tumor entities: leukemia (LE), lung cancer (LC), colon cancer (CO), cancer of the central nervous system (CNS), melanoma (ME), ovarian cancer (OV), renal cancer (RE), prostate cancer (PR) and breast cancer (BR).

This panel is frequently used in cancer research, and various studies have performed detailed analyses of the transcriptome and proteome of these cell lines to identify cancer-specific biomarkers or potential therapeutic targets. However, surface residing biomarkers might be missed by the latter approaches, since when employing standard-proteomics membranes are largely excluded in the non-soluble fraction and dynamic internalization processes of surface receptors, shedding and other mechanism that alter the receptorome are not detected by transcriptomics [15]. Furthermore, it remains unclear if biomarkers are also differentially expressed at the cell surface and may therefore be suitable targets for antibody-mediated immunotherapy. To close this important gap, we performed a medium-throughput flow cytometric profiling of the NCI-60 tumor cell panel with an arrayed set of 342 PE-labeled antibodies (Fig. 1 and Additional file 8). To probe for measurement reproducibility, 25 out of the 60 tumor cell lines were randomly selected for a subsequent measurement 1 week later, showing no significant differences (Spearman correlation coefficient r = 0.91, p < 2.2e−16) in the absolute mean fluorescence intensity (MFI)-values with same laser settings (Additional file 7). Importantly, data was matched to the corresponding isotype controls (IC) considering their background signal when calculating the true intensities. Detailed IC data is presented in Additional files 1, 2, 3, 4, 5 and 6,

### Multiomics analyses integrates flow cytometric data, proteomics and transcriptomics

It is important to note that differentially expressed receptors might in a first instance represent general tissue markers, as well as cancer specific markers. Hence, our subsequent workflow aims to screen for common markers first, eliminate those and then perform subfiltering for tissue and cancer specific markers.

To identify the most robustly expressed cell surface receptors on the various tumor cell lines, we integrated our FACS data with previously generated transcriptomic (Tx) and proteomic (Px) profiles of the NCI-60 tumor cell panel [13, 14, 19]. To this end, we used multiple co-inertia analysis (MCIA) to exploit the potential of the various omics data sets [19].

**Fig. 2:**
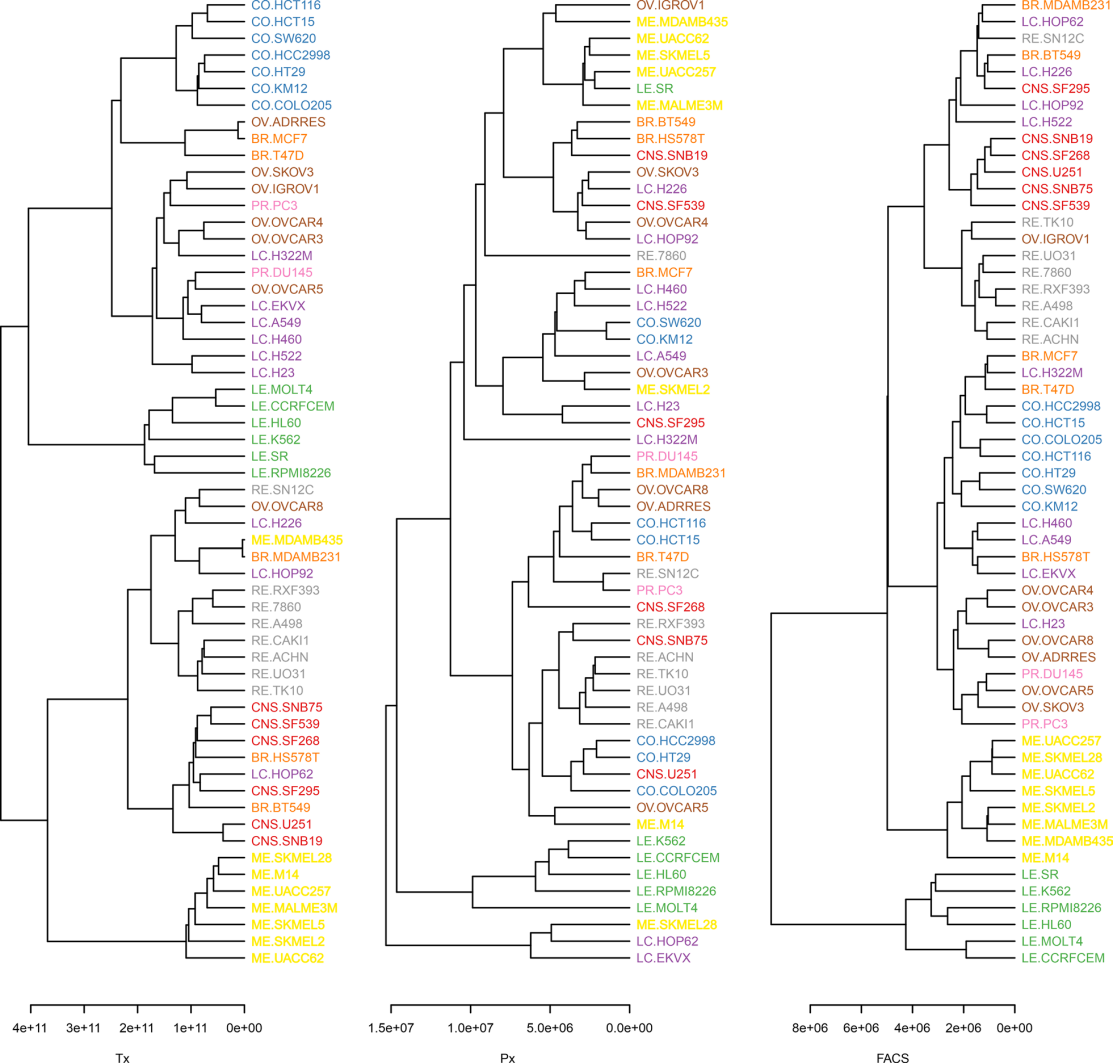
Tumor cell line relationship using different omics techniques. A dendrogram of Tx (left), Px (middle) and flow cytometric (right) data obtained from the 58 tumor cell lines from the NCI-60 tumor cell panel was build using Spearman distance calculation. Tumor cell lines were color coded according to the cancer entity they represent. Blue, colon cancer (CO); brown, ovarian cancer (OV); orange, breast cancer (BR); pink, prostate cancer (PR); purple; lung cancer (LC); green, leukemia (LE); grey, renal cancer (RE); red, cancer of the central nervous system (CNS); yellow, melanoma (ME)

During data curation, two cell lines had to be removed (see “Methods”), eventually ending up with 58 tumor cell lines, each yielding 332 features based on flow cytometry, 514 features from Px and 19,437 from Tx. Spearman distance was used to create a dendrogram of the various cell lines, visualizing their relationship and revealing potential differences in tumor cell line annotation upon utilization of different omics data (Fig. 2).

Of note, flow cytometry-based characterization, i.e., relationships between tumor cell lines based on expression of cell surface receptors, was comparable to the annotation based on Tx data, and superior to Px. Flow cytometric analysis revealed specific clusters of the respective cell lines, in detail of central nervous system, renal, melanoma, colon and leukemia tumor cell lines, indicating that these might harbor unique identifiers. For Tx, only lung cancer-derived tumor cell lines showed a more comprehensive clustering when compared to FACS, while ovarian, prostate and breast cancer cell lines were dispersed in all analyses (Fig. 2).

**Fig. 3:**
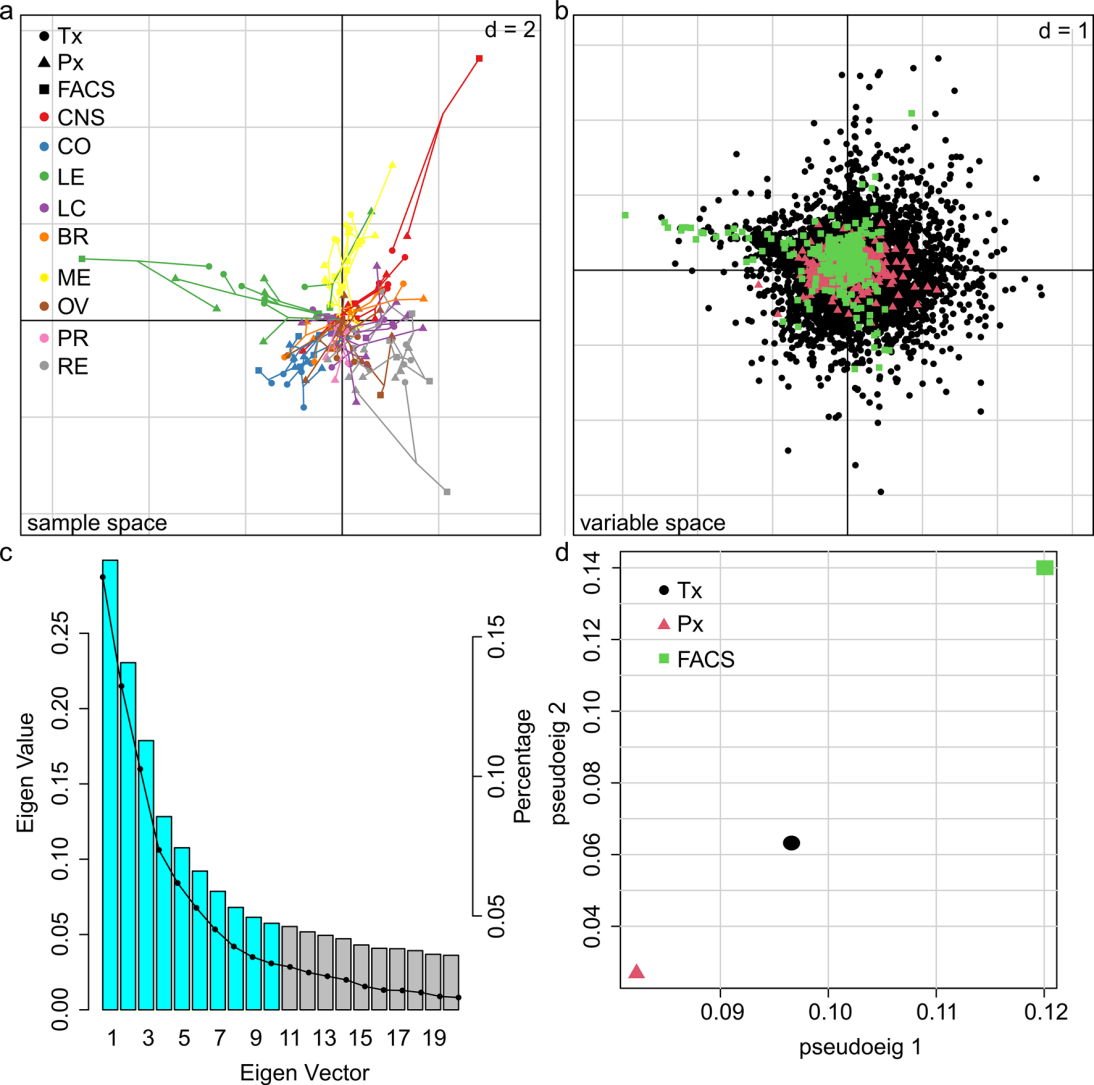
Multiple co-inertia analysis (MCIA) of the NCI-60 panel data. **a** (top left) The first two principal components of the MCIA plot show similar trends in microarray (Tx, circle), proteomics (Px, triangle), and flow cytometry (FACS, square) profiles, suggesting that the most variant sources of biological information are similar. The type of shape indicates the respective omics platform. Shapes are connected by lines joining a common point representing the maximized covariance reference structure derived from the MCIA analysis. The length of a line models the divergence between the data from the same tumor cell line. Colors represent the nine NCI-60 different tissues covered by the tumor cell lines. Central nervous system (CNS) and leukemia (LE) cell lines are separated along the first axis (PC1, horizontal). Melanoma (ME) was projected on the positive side of the second axis (PC2, vertical). **b** (top right) The variable space with data from the different omics techniques is colored coded (Tx, black; Px, red; FACS, green). A tissue specific feature will be projected in the direction of this tissue. The larger the distance from the origin, the more potentially significant a feature is. **c** (bottom left) A scree plot showing the eigenvalues on the y-axis and the number of PCs on the x-axis. Used to rationalize the number of PCs included in the analysis. **d** (bottom right) The pseudo-eigenvalue space of the NCI-60 data sets summarizes the consensus between the platforms, highlighting which omics technique contributes more to the total variance (Tx, black; Px, red; flow cytometry, green)

Comprehensive MCIA integrating all three omics approaches (Fig. 3) revealed distinct clusters for the individual cancer types (Fig. 3a). In fact, we observed a significant contribution of the flow cytometric surface proteome data to the molecular profile of the individual tumor cell lines. Furthermore, different tumor types showed similar molecular patterns, as they formed clusters within the sample space. In agreement to the relationship analyses based on the various single omics techniques (Fig. 2), the MCIA revealed unique signatures predominantly for melanoma (ME; yellow) and leukemia (LE; green), but also identifiable clusters for cancer of the central nervous system (CNS; red), renal cancer (RE; grey) and colon cancer (CO; blue) (Fig. 3a).

Figure 3b shows the variables (transcripts, proteins and surface receptors) and their contribution to the clustering. Moreover, scree plot analysis helped us to determine the number of factors that should be considered (Fig. 3c), hence the number of principal components (PCs) that contribute to variability and help to discriminate tumor entities. The first 10 PCs already account for 74.7% of the variance and therefore were included into MCIA. The pseudo-eigenvalues of the whole NCI-60 data set (Fig. 3d), including Tx, Px and flow-cytometry, demonstrates that the integration of the receptorome data crucially contributes to the total variance of the MCIA and is hence an essential denominator to identify biomarkers. Table 1 lists cell surface receptors that were identified as tissue specific biomarkers based on our MCIA. As anticipated, from the lack of clustering (Figs. 2 and 3a), no MCIA hits could be retrieved for lung-, breast, ovarian- and prostate cancer, which might also reflect the large heterogeneity of these cancer entities.

**Table 1:** Tumor cell line specific cell surface markers based on MCIA analysis

Colon cancer (CO)	Renal cancer (RE)	Melanoma (ME)	Central nervous system (CNS)	Leukemia (LE)	
CD104	CD106	CD1a	CD105	CD100	CD28
CD15	CD24	CD213a2	CD273B7DC	CD102	CD38
CD324	CD26	CD317	CD275B7H2	CD11a	CD4
CD326	EGFR	CD39	CD49e	CD18	CD45
CD49f	SSEA-3	CD49d	CD80	CD184	CD48
	SSEA-4	Integrin(a9b1)	MSCA1MSC	CD1b	CD5
	TIM1			CD1c	CD50
	TRA-1-60-R			CD1d	CD7
				CD2	CD84
				CD27	CD8a

### Identification of tumor biomarkers by integration of MCIA into recount2 RNA-seq data

As discussed, the cell surface receptors identified by the MCIA do not necessarily represent tumor biomarkers, as we are lacking healthy control tissue to compare with the tumor cell lines. As an example, leukocyte specific marker CD2 is a T cell antigen. Therefore, while it is not surprising that MCIA identified CD2 as a leukemia specific receptor in comparison to all the other cancer entities, it is conceivable that CD2 is not a tumor marker. Having now identified differentially expressed surface markers by FACS and MCIA, we would now ideally match the data with receptoromes of healthy control tissue. Due to the lack of such data, we validated our MCIA hits with The Cancer Genome Atlas (TCGA; www.cancer.gov/tcga) RNAseq count data, utilizing the recount2 resource results (https://jhubiostatistics.shinyapps.io/recount/ [32]). This allows to compare data from malignant with those from healthy tissues and hence to discriminate tissue markers from tumor markers. To our surprise, we could only retrieve for colon and renal cancer comprehensive recount2 RNAseq data from tumor as well as healthy tissues. For our other cancer types that distinctly clustered by MCIA, we could retrieve no TCGA RNAseq count data comparing tumor to normal tissue, precluding this type of analyses. The differentially expressed genes for colon and renal cancer are listed in Additional file 10: Dataset S3 and Additional file 11: Dataset S4. For both cancer entities, the five (colon) and eight (renal) identified MCIA receptors were differentially expressed between healthy and malignant tissues and hence represent potential surface accessible tumor biomarkers (Table 2).

**Table 2:** CO and RE cancer cell surface biomarkers based on integrated MCIA and recount2

log2FoldChange	pvalue	padj	receptor ID	Gene ID
Colon cancer: recount2 analysis filtered by MCIA hits				
0.659	6.844e−10	3.064e−9	CD49f	ITGA6
0.503	1.923e−06	6.064e−06	CD15	FUT4
0.328	0.016	0.029	CD104	ITGB4
− 0.431	3.118e−06	9.609e−06	CD326	EPCAM
− 0.489	2.994e−8	1.144e−7	CD324	CDH1
Renal cancer: recount2 analysis filtered by MCIA hits				
2.304	1.021e−36	1.237e−35	TIM1	HAVCR1
1.726	4.503e−36	5.309e−35	CD106	VCAM1
1.464	7.690e−63	2.500e−61	SSEA-4	TMCC1
1.371	6.499e−11	2.119e−10	SSEA-3	B3GALT5
1.200	1.296e−32	1.323e−31	EGFR	EGFR
0.681	9.772e−7	2.383e−06	CD26	DPP4
0.464	1.575e−8	4.375−8	CD24	CD24
− 1.856	7.622e−41	1.1002e−39	TRA-1-60-R	PODXL

**Fig. 4:**
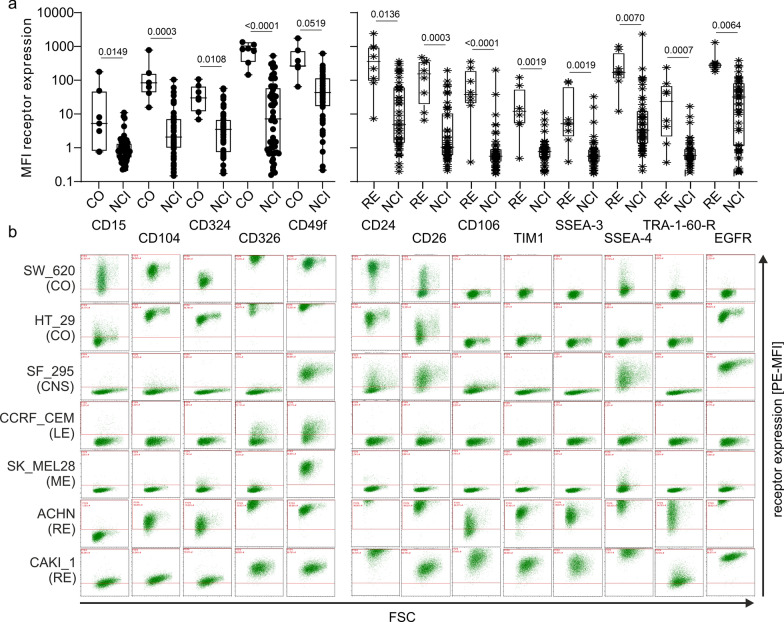
Differential cell surface receptor expression on CO and RE cancer cell lines as compared to other tumor cell lines. **a** Shown is the relative MFI of each of the depicted receptors of each CO (n = 7, left) or RE (n = 8, right) cell line in comparison to all other tumor cell lines in the NCI-60 panel (NCI). Each symbol represents the specific cell surface receptor expression MFI of one cell line. Statistical significance of overall differences in receptor expression (MFI) of CO or RE versus all other cell lines was calculated with a one-way ANOVA with multiple comparisons and a Kruskal–Wallis test assuming non-gaussian distribution. **b** Primary FACS plots from representative cell lines of the various tumor entities. Plotted is the size of the cells (forward scatter, FSC) vs. the relative fluorescence expression of the respective cell surface marker (PE, mean fluorescence intensity, MFI). Note that no representative FACS plots are shown for ovarian cancer (OV), breast cancer (BR), prostate cancer (PR) and lung cancer (LC) because these cancer entities did not form clusters based on flow cytometric receptor expression (compare Fig. 2)

We next plotted the relative MFIs of the tumor biomarkers and directly compared receptor expression on colon and renal tumor cell lines in the NCI-60 tumor cell panel to the other cell lines (Fig. 4). All receptors showed increased expression on colon and renal cancer cell lines as compared to the remaining tumor cell lines from the NCI-60 panel. Expression of CD15, CD104, CD324, CD326 and CD49f was specifically enriched on the surface of colon cancer-derived cell lines (Fig. 4a, left and primary FACS data in in Fig. 4b). Renal cancer cell lines in comparison to all other cancer entities expressed significantly higher cell surface levels of CD24, CD26, CD106 (VCAM1), TIM1, SSEA-3 (B3GALT5), SSEA-4 (TMCC1), TRA-1-60R (PODXL) and EGFR (Fig. 4a right and primary FACS data in Fig. 4b).

### Immunohistochemical analysis and validation of kidney tumor biomarkers using the human protein atlas

**Fig. 5:**
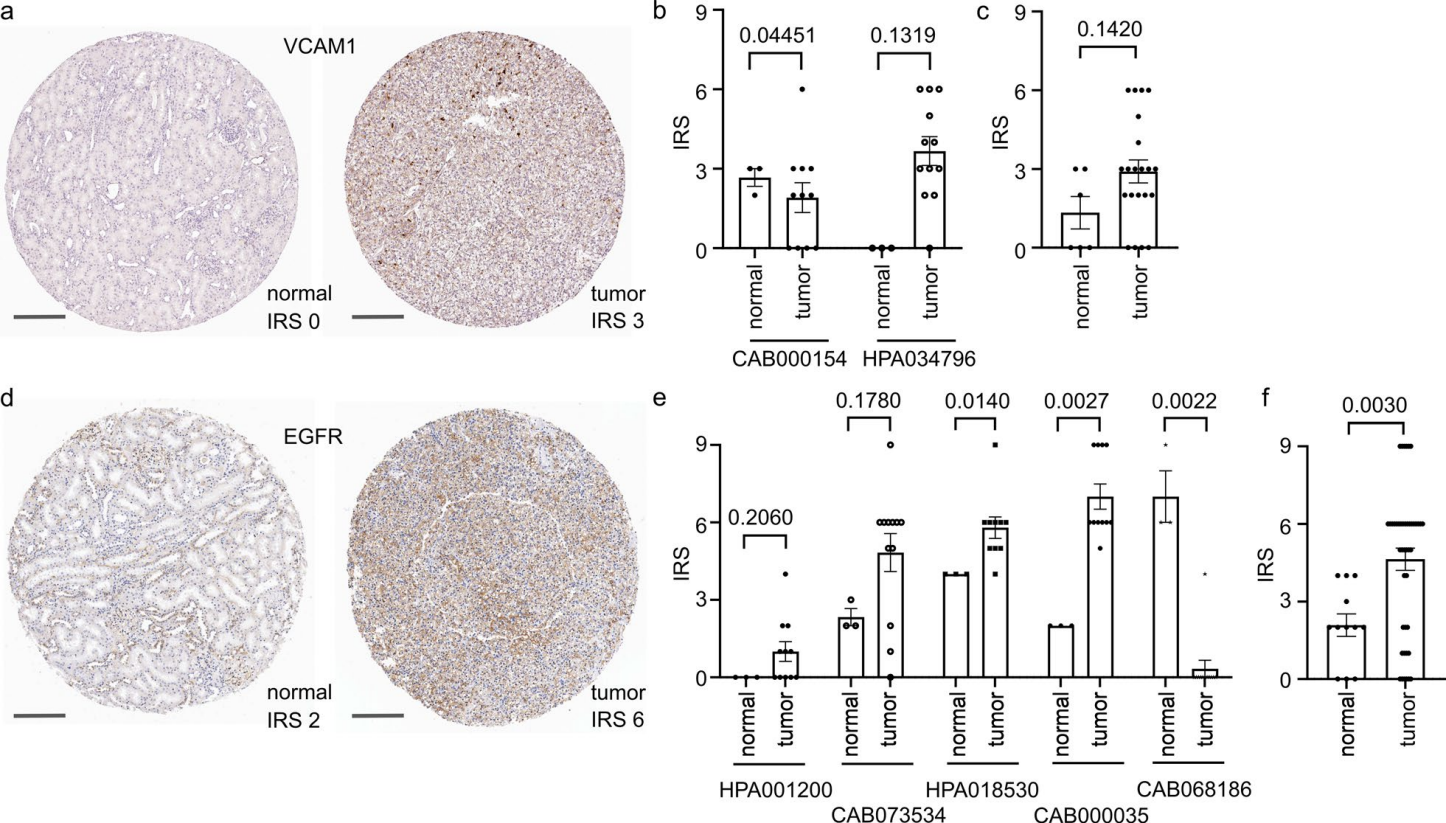
Expression of VCAM1 and EGFR in healthy vs. tumor tissue based on HPA data. **a** Representative tissue cores from the HPA showing immunohistochemistry (IHC) staining for VCAM1. Left core, normal kidney; right core, renal cancer. **b**, **c** Summarized data for immunoreactive scores (IRS) for normal (pooled n = 6) and tumor kidney (pooled n = 23) from two antibodies shown separately (**b**) and pooled (**c**). **d** Representative tissue cores from the HPA showing IHC staining for EGFR. Left core, normal kidney; right core, renal cancer. **e**, **f** Summarized data for IRS for normal (pooled n = 12) and tumor kidney (pooled n = 44) from five antibodies shown separately (**e**) and pooled (**f**). In **f**, antibody CAB068186 which showed opposite results was not included. Scale bars, 200 μm

We next addressed the expression of kidney tumor biomarkers at the protein level by using data from the Human Protein Atlas (HPA, www.proteinatlas.org, [34]). CD106 (VCAM1) protein expression tended to be lower in healthy renal tubules compared to renal adenocarcinoma (Fig. 5a–c). For EGFR, we observed the same trend with higher expression in the tumors (Fig. 5d, e). whereas one EGFR antibody clone had the opposite phenotype (Fig. 5e). Pooled analysis without that clone showed a significantly higher protein expression of EGFR in tumors compared to healthy tubules (p = 0.0030; exact, two-tailed; sum of ranks 199.5, 1397; Mann–Whitney U = 121.5; Fig. 5f). There were no significant differences in expression between clear cell and non-clear cell renal cell carcinomas for CD106 (n = 23; p = 0.787; df = 5, value = 2.428, chi-squared test) and EGFR (n = 44; without CAB068186 antibody; p = 0.432; df = 6; value = 5.919, chi-squared test).

 For CD24, we also observed moderately higher protein levels in tumors, which is depicted in Additional file 12: Fig. S7. For TRA-1-60R (PODXL), there were no significant differences detected (Additional file 12, panel d, e). Interestingly, and in contrast to the expected outcomes from our mRNA analysis, we observed significantly lower expression levels of TIM1 (HAVCR1), SSEA4 (TMCC1), CD26 and SSEA3 (B3GALT5) in tumors compared to healthy renal tubules (Additional file 12, panel f–o). The whole data are summarized in Additional file 8: Table S1.

Hence, while previous studies including data summarized in the HPA provided a list of promising candidates potentially suitable to use as cancer biomarkers, our data now critically expands this knowledge to markers that are surface accessible and are therefore candidates for tumor specific immunotherapy. Especially CD106 (VCAM1) and EGFR seem promising immunotherapeutic targets that show higher surface expression levels in the context of renal cancer.

### Receptor expression of kidney cancer subtypes by functional proteomics

Reverse Phase Protein Array (RPPA) data of the “The Cancer Proteome Atlas” (TCPA, https://tcpaportal.org/tcpa/, [36]) was assessed in order to explore the EGFR functional proteomics landscape of all renal cell cancer subtypes evaluated in this portal: These are “Kidney Chromophobe” (KICH, n = 63), “Kidney renal clear cell carcinoma” (KIRC, n = 445), and “Kidney renal papillary cell carcinoma” (KIRP, n = 208). Since CD106 (VACM1) and EGFR seemed to be promising targets based on our previous analysis we checked the dataset for these two markers. No data for VCAM1 was found. For EGFR, KICH and KIRP were compared against KIRC, since KIRC is the most frequent renal cancer subtype. Both comparisons revealed a significant difference when comparing the expression levels of EGFR (Table 3).

**Table 3:** Kidney cancer (RE) subtype specific expression analysis of EGFR via functional proteomics (TCPA)

Comparison	Expression A	Expression B	pvalue
KIRC vs. KICH	0.74176	0.32398	1.2851e−10
KIRC vs. KIRP	0.74176	0.21612	1.8623e−72

This result is in slight contrast to HPA (Fig. 5), by which we did not find kidney cancer subtype specific differences in EGFR expression (Fig. 5). This is most likely due to the much smaller sample size in HPA with only nine non-clear cell renal cell carcinoma. More importantly, the TCPA analysis provides an independent confirmation of the high expression of EGFR in renal cell cancer and further indicates that EGFR could be a subtype specific biomarker for kidney cancer.

## Discussion

By employing a comprehensive flow cytometric screen and combined multiomics analyses (integrating previously defined Tx and Px datasets), we identified novel specific cell surface expression patterns in five of the nine cancer entities represented by the NCI-60 tumor cell panel.

In the first instance, these receptors do not represent tumor biomarkers, as their identification is based on comparisons within the NCI-60 tumor cell panel. However, by subsequent cross-analysis with the TCGA recount2 RNAseq data, which also comprises healthy tissue for comparison [32], we could deconvolute our data to identify tumor specific biomarkers for two tumor entities, i.e., colon and renal cancer.

In theory, MCIA of the NCI-60 tumor cell panel with Tx and Px data alone, followed by annotation of biomarkers to their specific localization, also could have enabled the identification of cell surface specific receptor expression. However, implementation of the flow cytometric screening data provides several advantages. First, it is clear from the MCIA that integration of the flow cytometric data critically expands the sample space and strongly contributes to the overall variation (Fig. 3). Second, by employing flow cytometry, which is an antibody-based detection method, we already pre-screen for surface markers that can be detected and targeted by antibodies, anticipating a subsequent exploitation of these receptors for both diagnostic and immunotherapeutic (i.e., theranostic) applications. On the other hand, combining flow cytometry with the already available Tx and Px data by MCIA strongly enhanced confidence in our hits.

The initial MCIA revealed specific cell surface markers allowing to discriminate colon cancer (CO), melanoma (ME), renal cancer (RE), cancer of the central nervous system (CNS) and leukemia (LE) cancer cell lines from all other cell lines within the NCI-60 tumor cell panel (Table 1). On the other hand, for the other four cancer entities, i.e., lung cancer (LC), breast cancer (BR), ovarian cancer (OV) and prostate cancer (PR) we failed to annotate a specific cell surface marker expression pattern, which might be due to the large heterogeneity of these tumor types.

We were surprised about our difficulties in finding accessible and reliable data to compare our MCIA derived potential tumor biomarkers with healthy tissue. Only for healthy colon and renal tissue we succeeded to extract healthy tissue RNAseq count data from the recount2 data base, allowing to cross-validate our cancer cell line-derived markers for differential expression between malignant and healthy tissue. Direct investigation of the TCGA portal revealed that healthy tissue RNAseq count data for skin, bone marrow, and lymph nodes is missing. Hence, further work to obtain omics data from healthy tissues in case of melanoma (ME), cancer of the central nervous system (CNS) and leukemia (LE) is warranted to obtain tumor markers for the latter tissues, too.

Ultimately, based on our MCIA, we identified CD15, CD104 (Integrin-β4), CD324 (E-cadherin), CD326 (EpCAM), and CD49f as biomarkers for colon cancer and CD24, CD26 (DPP4), CD106 (VCAM1), TIM-1, SSEA-3 (B3GALT5), SSEA-4 (TMCC1), TRA-1-60-R (PODXL) and EGFR for renal cancer. Of note, the colon cancer biomarkers identified by our approach have been proposed as potential tumor markers in colorectal cancer before [37–43]. These findings raise confidence in our data and independently confirm the stringency in our experimental screening and MCIA on the one hand, but also suggest following up on these receptors as structures for tumor-targeted immunotherapy. Similarly, for renal cancer, EGFR and TIM-1 are established tumor biomarkers for which immunotherapy has been proposed [44–46] and a phase I clinical trial with the goal to treat renal cell carcinoma with a TIM-1 targeting antibody indicated efficacy with manageable adverse effects [47]. Beyond that, CD24 was also proposed as a renal cancer biomarker [48, 49] and is discussed as a “hot candidate” for targeted immunotherapy, as it emerged as a novel “don’t eat me”-signal that is expressed on various tumors and prevents their phagocytosis by macrophages [50, 51]. The role of CD26 is less explored in renal cancer even though it is suggested as a target for immunotherapy in the context of other cancer entities including the generation of CD26 directed CAR-T cells [52–56]. There is less experimental evidence establishing the other receptors identified by us as renal cancer-specific biomarkers. SSEA-3 and SSEA-4 as well as TRA-1-60-R are described as stem cell markers with some associations to renal cancer, that might be linked with aggressive tumor progression in vivo [57–61].

Likewise, CD106 (VCAM1) might be an interesting and potential novel cell surface biomarker as well as an attractive target for immunotherapy in the context of renal cancer. Currently, the role of VCAM1 in renal cancer is less explored, with data pointing towards protective roles in this cancer entity [62], as well as a potential involvement of VCAM1 in tumor immune evasion [63]. Furthermore, VCAM1 plays an important role in anti-tumor T cell responses and T cell infiltration into tumors [64, 65]. For VCAM1, EGFR and CD24 our findings were validated with healthy and tumor tissue data obtained from the Human Protein Atlas. This set of data provides further independent indications that VCAM1, EGFR and CD24 are highly expressed in renal cancers and that their high expression is a poor prognostic marker for survival [49, 66]. Given that EGFR and CD24 are already proposed and in the process of being therapeutically exploited, we now propose VCAM1 as a novel biomarker and target for cancer-specific stratified immunotherapy.

Furthermore, “The Cancer Proteome Atlas” resource enabled us to investigate kidney cancer subtype specific expression of EGFR, indicating that this receptor is a specific biomarker for clear cell renal cell carcinoma.

## Conclusion

Altogether, our work adds a comprehensive panel of potential surface accessible cancer biomarkers which need to be further characterized by mining of databases and being evaluated in primary patient tumor tissue and healthy control tissues. Thus, our distinct approach demonstrates the power of open data integration and open science for fundamental and translational research. Furthermore, efforts to develop immunotherapeutic approaches utilizing these biomarkers, as for instance CAR T cells and bispecific antibodies for specific and direct elimination of these tumors are highly important and warranted.

## Supplementary Information

**Additional file 1: Figure S1.** Boxplot showing MFIs of all Isotype antibody controls. 

**Additional file 2: Figure S2.** Boxplot showing MFIs of all mouse Isotype antibody controls.

**Additional file 3: Figure S3.** Boxplot showing MFIs of all rat Isotype antibody controls.

**Additional file 4: Figure S4.** Boxplot showing MFIs of AHIgGITCL Isotype antibody controls.

**Additional file 5: Figure S5.** Boxplot showing MFIs of all Mouse antibodies without Mouse IgG3 antibodies.

**Additional file 6: Figure S6.** Boxplot showing MFIs of Mouse IgG3 antibodies only.

**Additional file 7: Dataset S1.** Correlation antibody staining, dataset showing the correlation in antibody staining from two different biological replicates in 2 subsequent weeks.

**Additional file 8: Dataset S2.** Dataset including all mean fluorescence intensity values (MFIs] from all FACS screens done with the NCI-60 panel.

**Additional file 9: Table S1.** Table summarizing the results of the HPA analysis related to the biomarkers identified for renal cancer.

**Additional file 10: Dataset S3.** Dataset summarizing the analysis of the Recount2 RNAseq data analysis for differential RNA levels in healthy versus tumor colon tissue.

**Additional file 11: Dataset S4.** Dataset summarizing the analysis of the Recount2 RNAseq data analysis for differential RNA levels in healthy versus tumor renal tissue.

**Additional file 12: Figure S7.** This figure shows the results of the analysis of biomarker expression in normal and tumor kidney by immunohistochemistry based on HPA data.

## Data Availability

All data generated and analyzed during this study are included in this published manuscript. The analysis scripts are available at https://github.com/qbicsoftware/QMSFC.
