# Supplementary figures and images for: Multiomics surface receptor profiling of the NCI-60 tumor cell panel uncovers novel theranostics for cancer immunotherapy
Source: Cancer Cell Int. 2022 Oct 11;22:311. doi: 10.1186/s12935-022-02710-y (PMC9555072; doi:10.1186/s12935-022-02710-y)

MFI values of IsotypeControl All

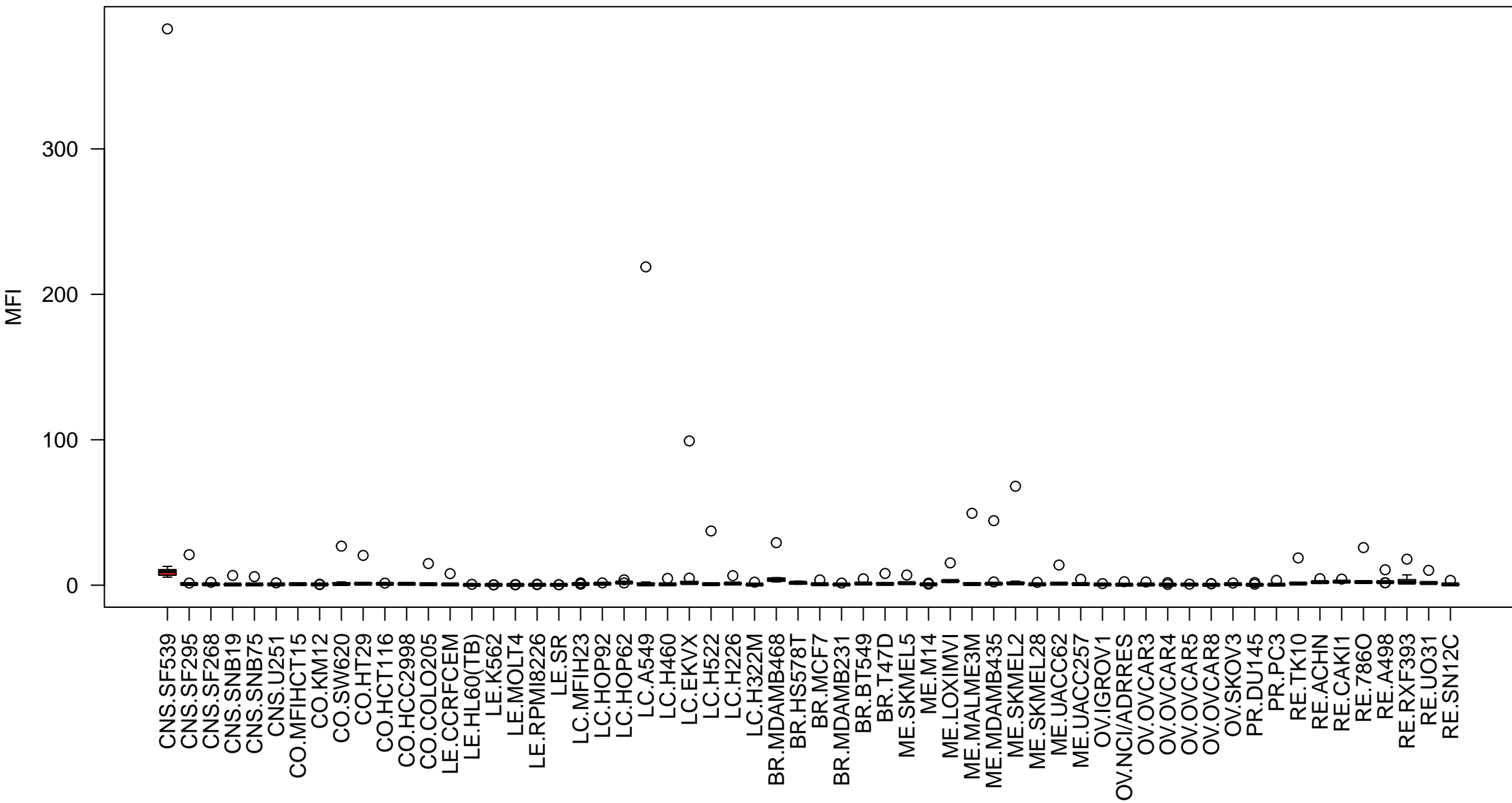

Supplement: Supplementary file 1 — Additional file 1: Figure S1. Boxplot showing MFIs of all Isotype antibody controls. [file 12935_2022_2710_MOESM1_ESM.pdf]

MFI values of IsotypeControl Mouse Only

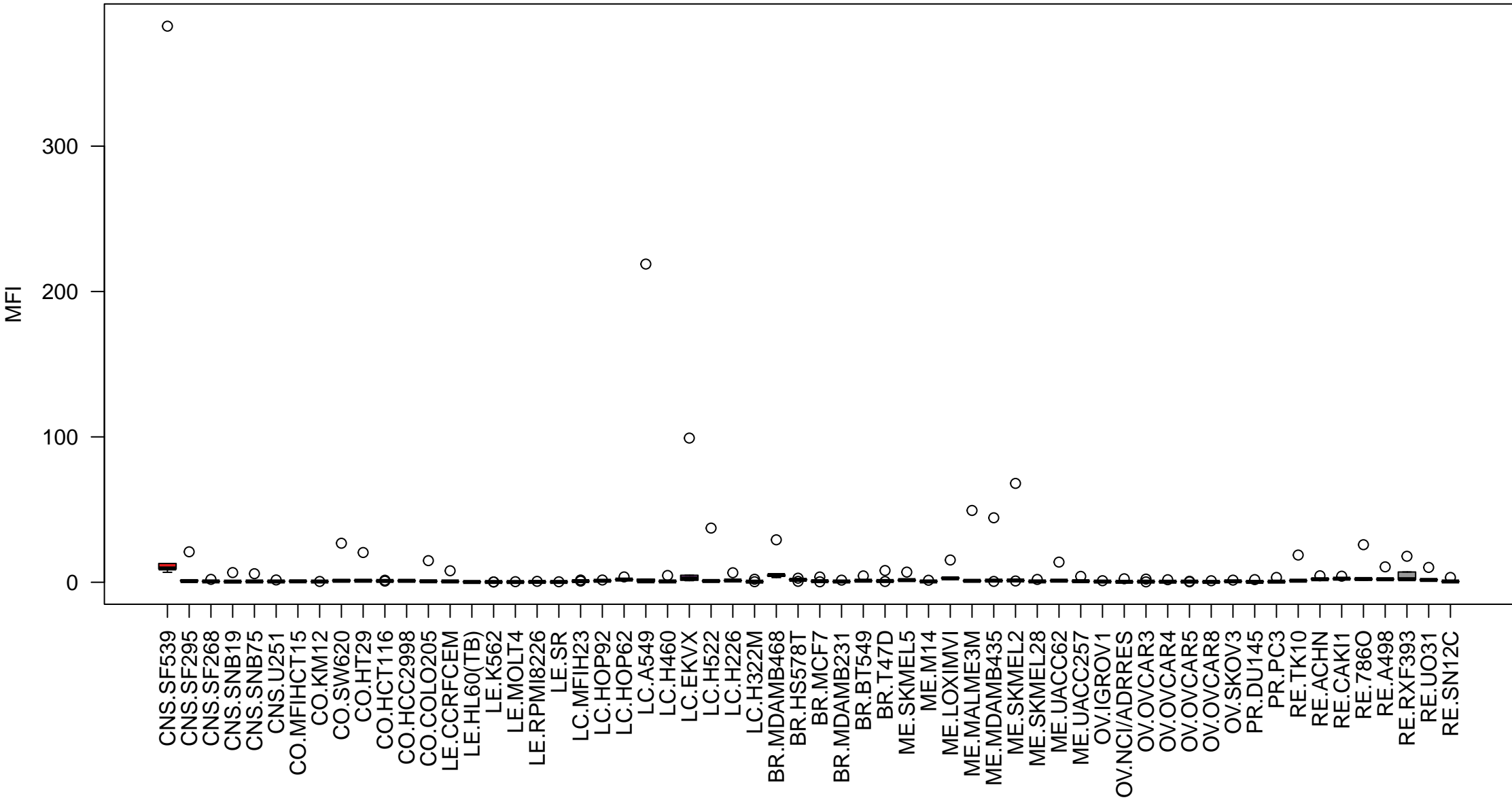

Supplement: Supplementary file 2 — Additional file 2: Figure S2. Boxplot showing MFIs of all mouse Isotype antibody controls. [file 12935_2022_2710_MOESM2_ESM.pdf]

MFI values of IsotypeControl Rat Only

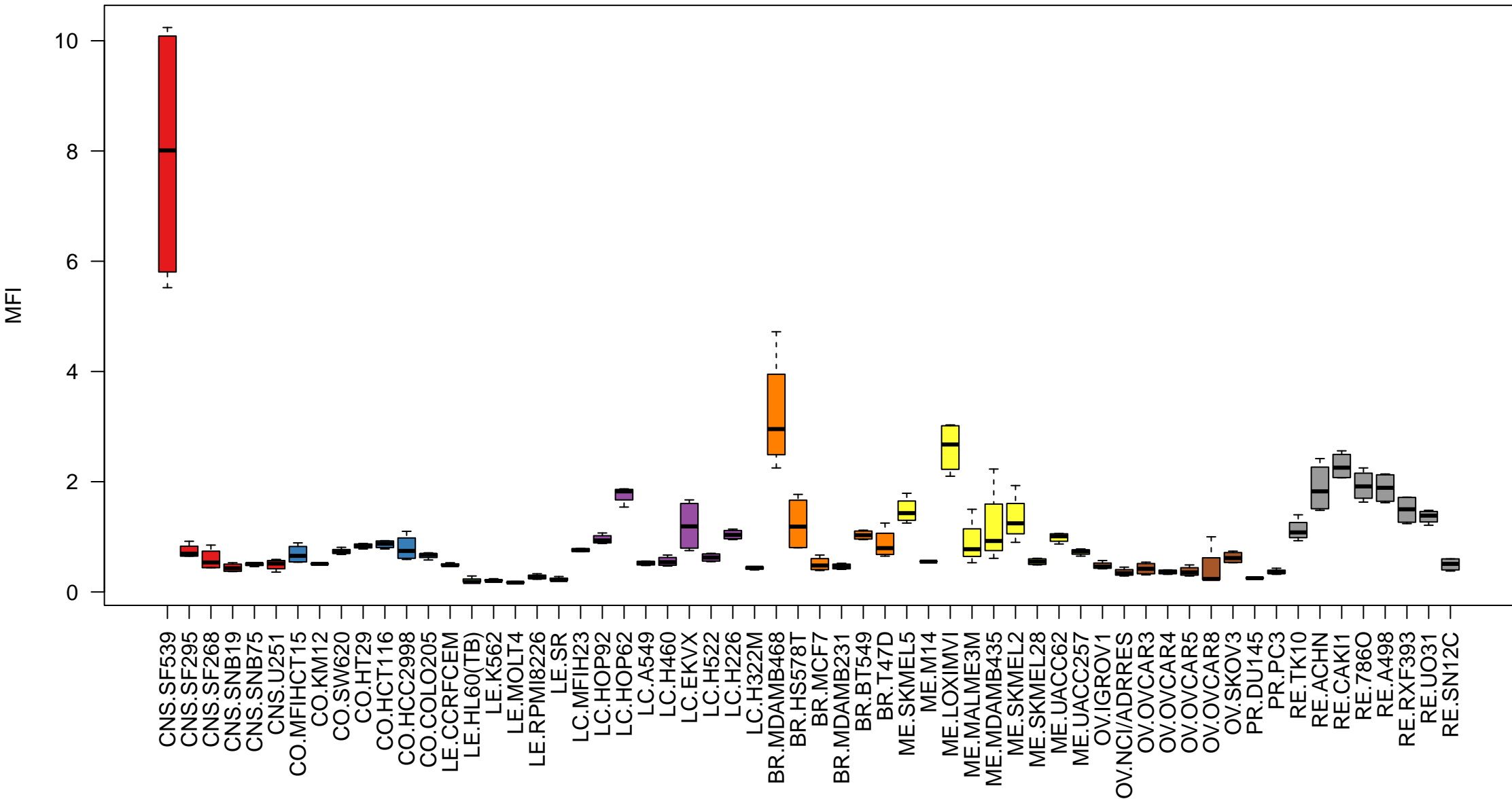

Supplement: Supplementary file 3 — Additional file 3: Figure S3. Boxplot showing MFIs of all rat Isotype antibody controls. [file 12935_2022_2710_MOESM3_ESM.pdf]

MFI values of IsotypeControl AHIgGITCL Only

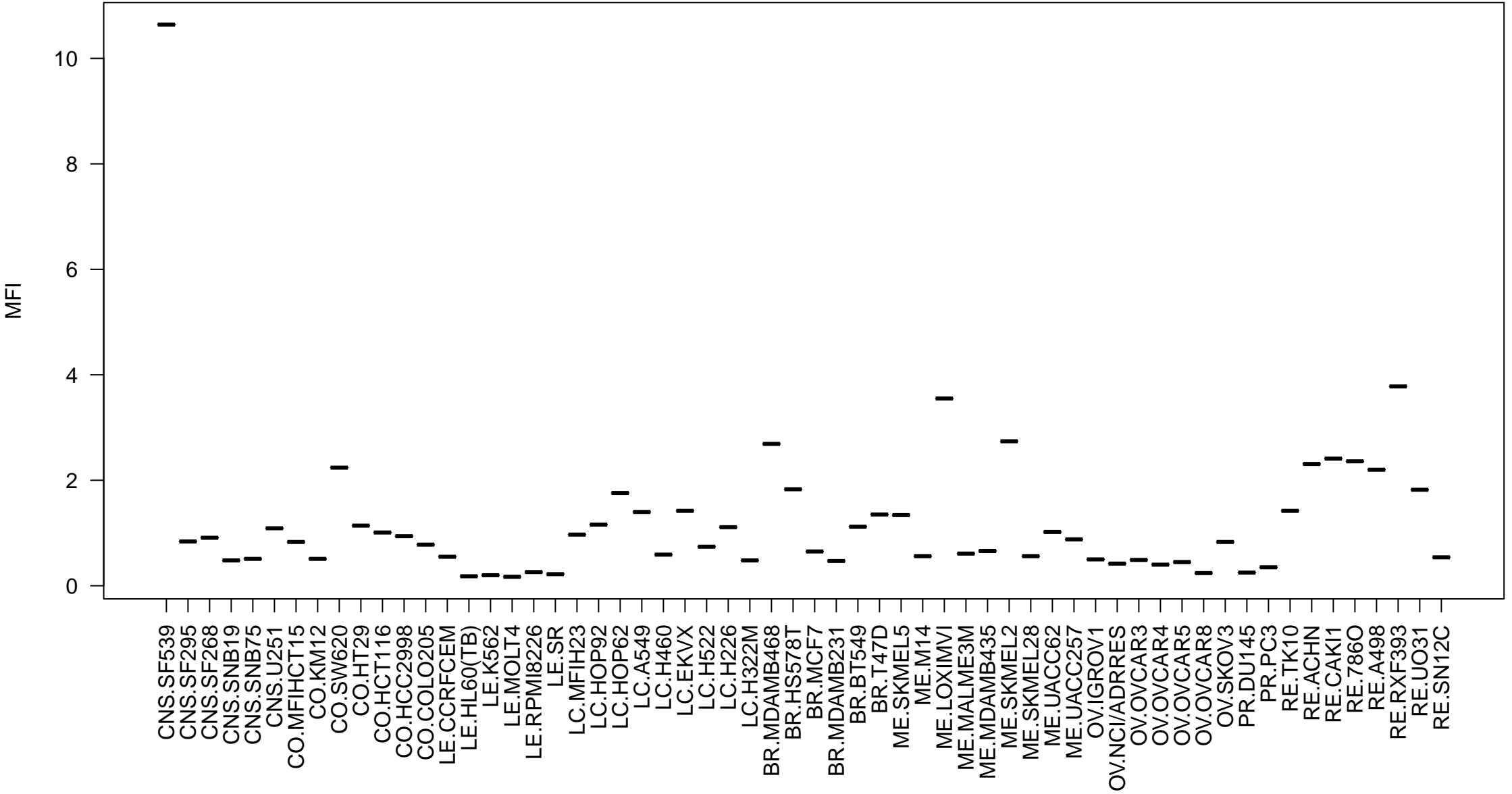

Supplement: Supplementary file 4 — Additional file 4: Figure S4. Boxplot showing MFIs of AHIgGITCL Isotype antibody controls. [file 12935_2022_2710_MOESM4_ESM.pdf]

MFI values of IsotypeControl Mouse without MslgG3kappaITCL

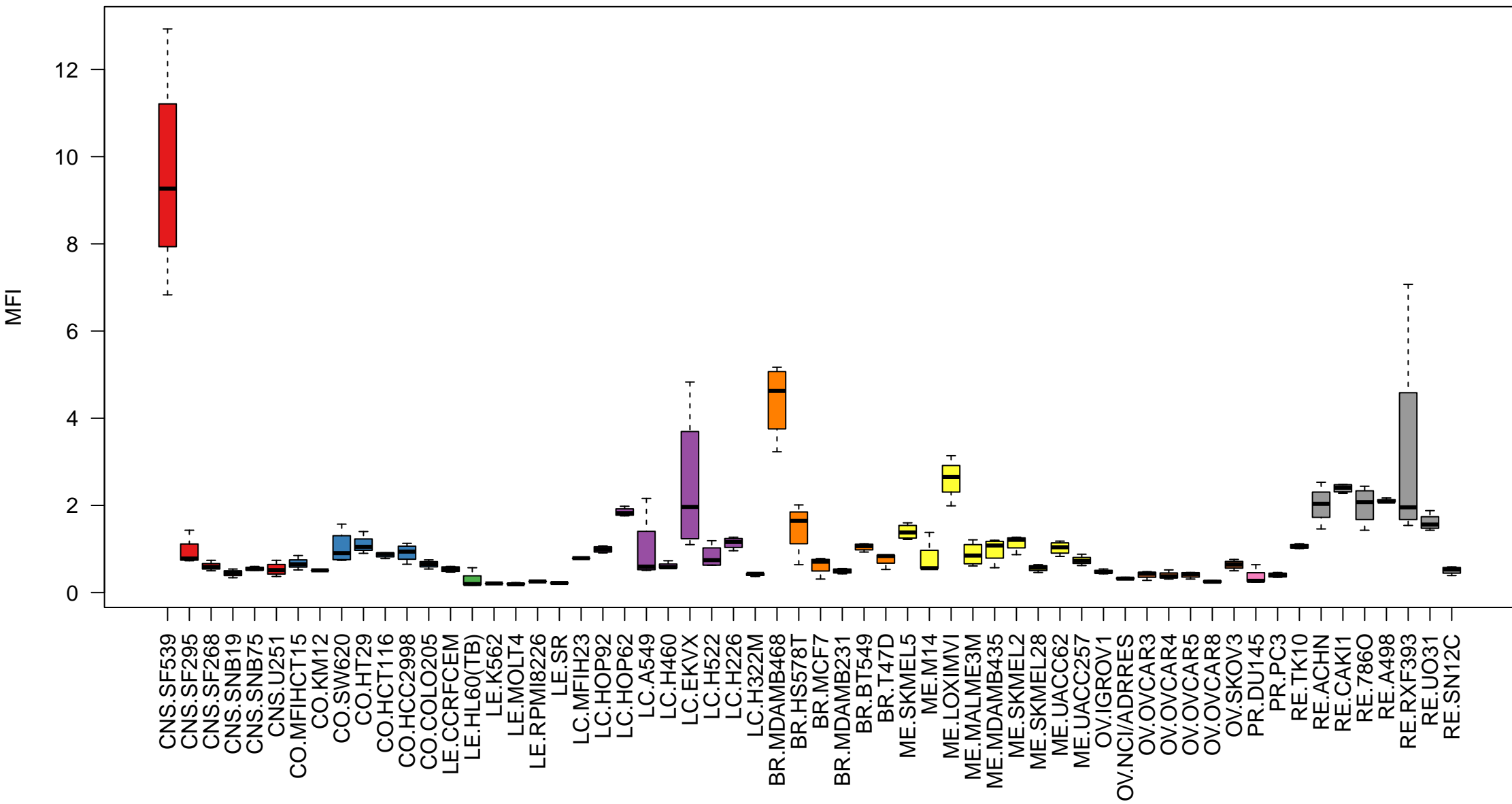

Supplement: Supplementary file 5 — Additional file 5: Figure S5. Boxplot showing MFIs of all Mouse antibodies without Mouse IgG3 antibodies. [file 12935_2022_2710_MOESM5_ESM.pdf]

MFI values of IsotypeControl Mouse MslgG3kappaTCL Only

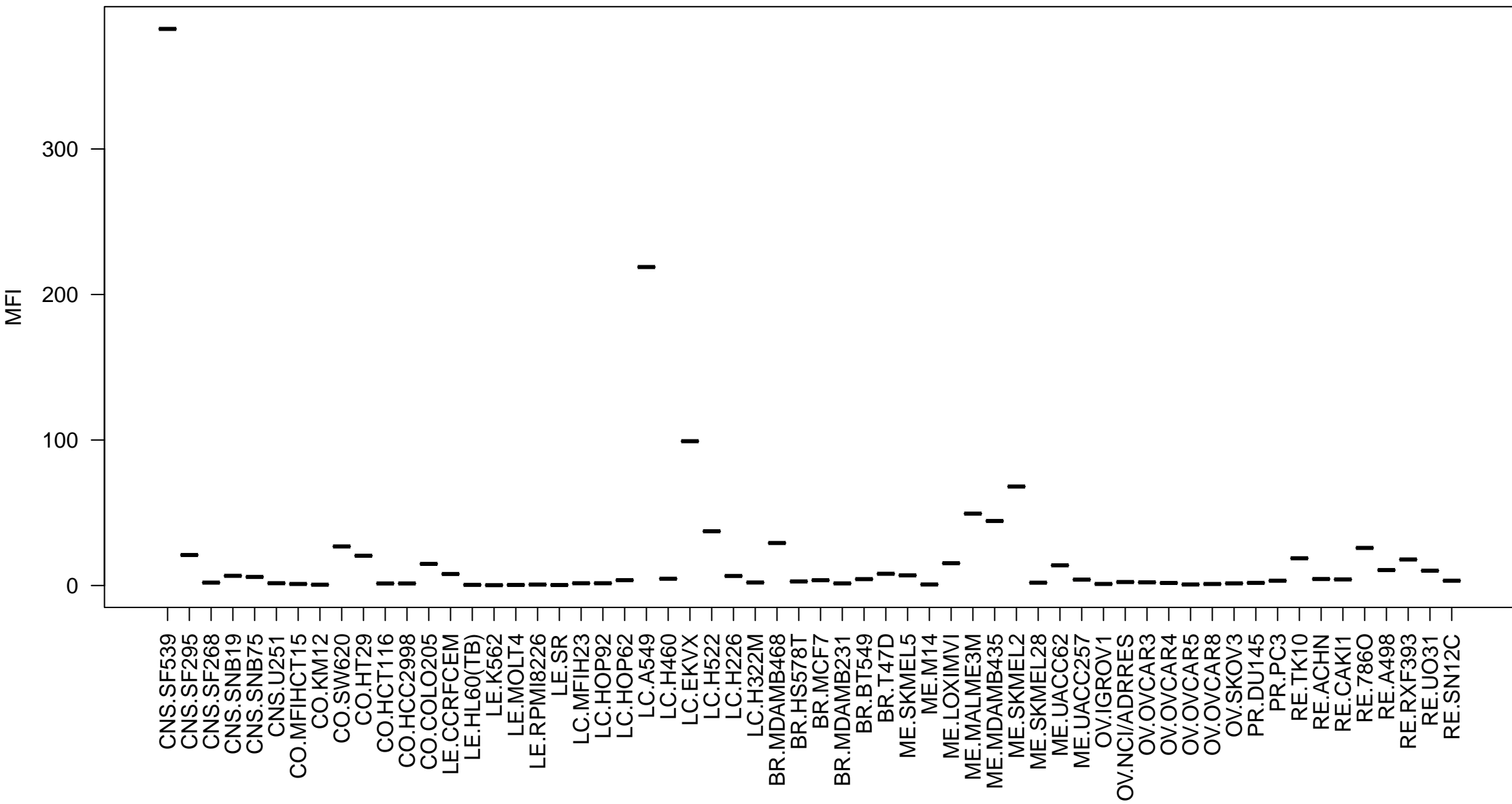

Supplement: Supplementary file 6 — Additional file 6: Figure S6. Boxplot showing MFIs of Mouse IgG3 antibodies only. [file 12935_2022_2710_MOESM6_ESM.pdf]

Correllation test of all cell lines week 1 versus week 2

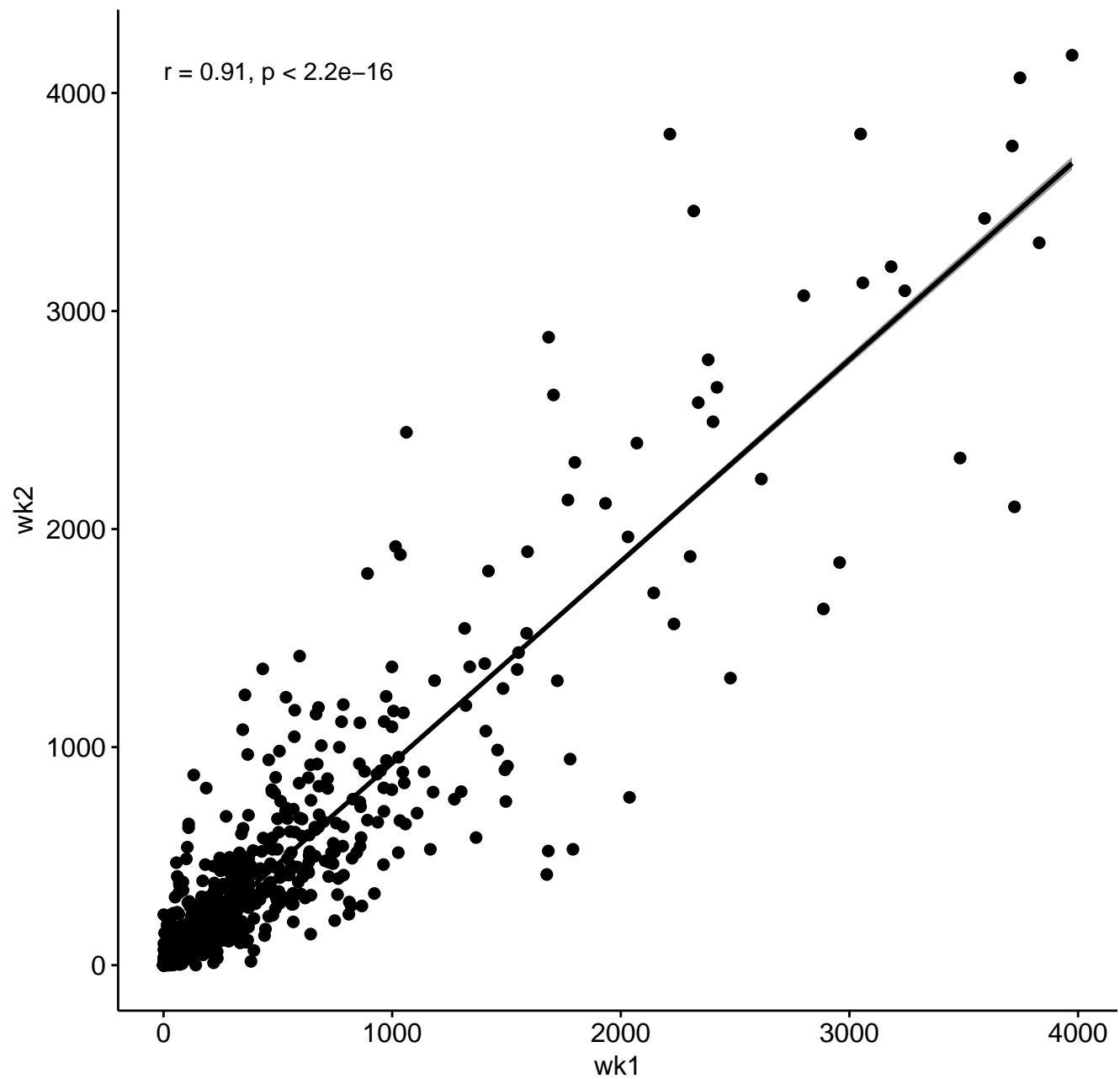

Supplement: Supplementary file 7 — Additional file 7: Dataset S1. Correlation antibody staining, dataset showing the correlation in antibody staining from two different biological replicates in 2 subsequent weeks. [file 12935_2022_2710_MOESM7_ESM.pdf]
