# Supplementary material for: Multiomics surface receptor profiling of the NCI-60 tumor cell panel uncovers novel theranostics for cancer immunotherapy
Source: Cancer Cell Int. 2022 Oct 11;22:311. doi: 10.1186/s12935-022-02710-y (PMC9555072; doi:10.1186/s12935-022-02710-y)
Supplement: Supplementary file 9 — Additional file 9: Table S1. Table summarizing the results of the HPA analysis related to the biomarkers identified for renal cancer. [file 12935_2022_2710_MOESM9_ESM.docx]

| **Molecule** | Antibody | n patients  (healthy kidney) | IRS mean (range)  healthy kidney | n patients  (neoplastic kidney) | IRS mean (range) neoplastic kidney |
| --- | --- | --- | --- | --- | --- |
| **CD106 (VCAM1)** | HPA034796  CAB000154 | n=3  n=3 | 0  2.7 (2 to 3) | n=12  n=11 | 3.7 (0 to 6)  1.9 (0 to 3) |
| **EGFR** | HPA001200  CAB073534  HPA018530  CAB000035  CAB068186 | n=3  n=3  n=3  n=3  n=3 | 0  2.3 (2 to 3)  4 (4)  2 (2)  7 (6 to 9) | n=11  n=12  n=10  n=11  n=12 | 1 (0 to 4)  4.8 (0 to 9)  5.8 (4 to 9)  7 (5 to 9)  0.3 (0 to 4) |
| **TIM1 (HAVCR1)** | CAB075697 | n=3 | 6 (6) | n=12 | 3.2 (1 to 6) |
| **SSEA4 (TMCC1)** | HPA053894 | n=3 | 4 (3 to 6) | n=11 | 0.6 (0 to 3) |
| **SSEA3 (B3GALT5)** | HPA054092  HPA054684 | n=3  n=3 | 6 (6)  1.3 (0 to 2) | n=11  n=11 | 2.1 (0 to 6)  0.4 (0 to 2) |
| **CD26 (DPP4)** | HPA068778  HPA071236  CAB045970 | n=3  n=3  n=3 | 9 (9)  9 (9)  8 (6 to 9) | n=12  n=11  n=12 | 5.8 (0 to 9)  4.6 (0 to 9)  3.6 (0 to 9) |
| **CD24** | CAB078471 | n=3 | 4 (3 to 6) | n=12 | 5.5 (1 to 6) |
| **TRA-1-60R (PODXL)** | HPA002110  CAB068220  CAB016169  CAB068219  CAB062558  HPA045507 | n=3  n=2  n=3  n=1  n=3  n=2 | 2.7 (2 to 4)  0  0  2  0.7 (0 to 2)  0 | n=12  n=11  n=11  n=11  n=12  n=11 | 1.6 (0 to 6)  0.5 (0 to 3)  0.2 (0 to 2)  0.3 (0 to 2)  0  0 |
