# Supplementary material for: Multiomics surface receptor profiling of the NCI-60 tumor cell panel uncovers novel theranostics for cancer immunotherapy
Source: Cancer Cell Int. 2022 Oct 11;22:311. doi: 10.1186/s12935-022-02710-y (PMC9555072; doi:10.1186/s12935-022-02710-y)
Supplement: Supplementary file 12 — Additional file 12: Figure S7. This figure shows the results of the analysis of biomarker expression in normal and tumor kidney by immunohistochemistry based on HPA data. [file 12935_2022_2710_MOESM12_ESM.pdf]

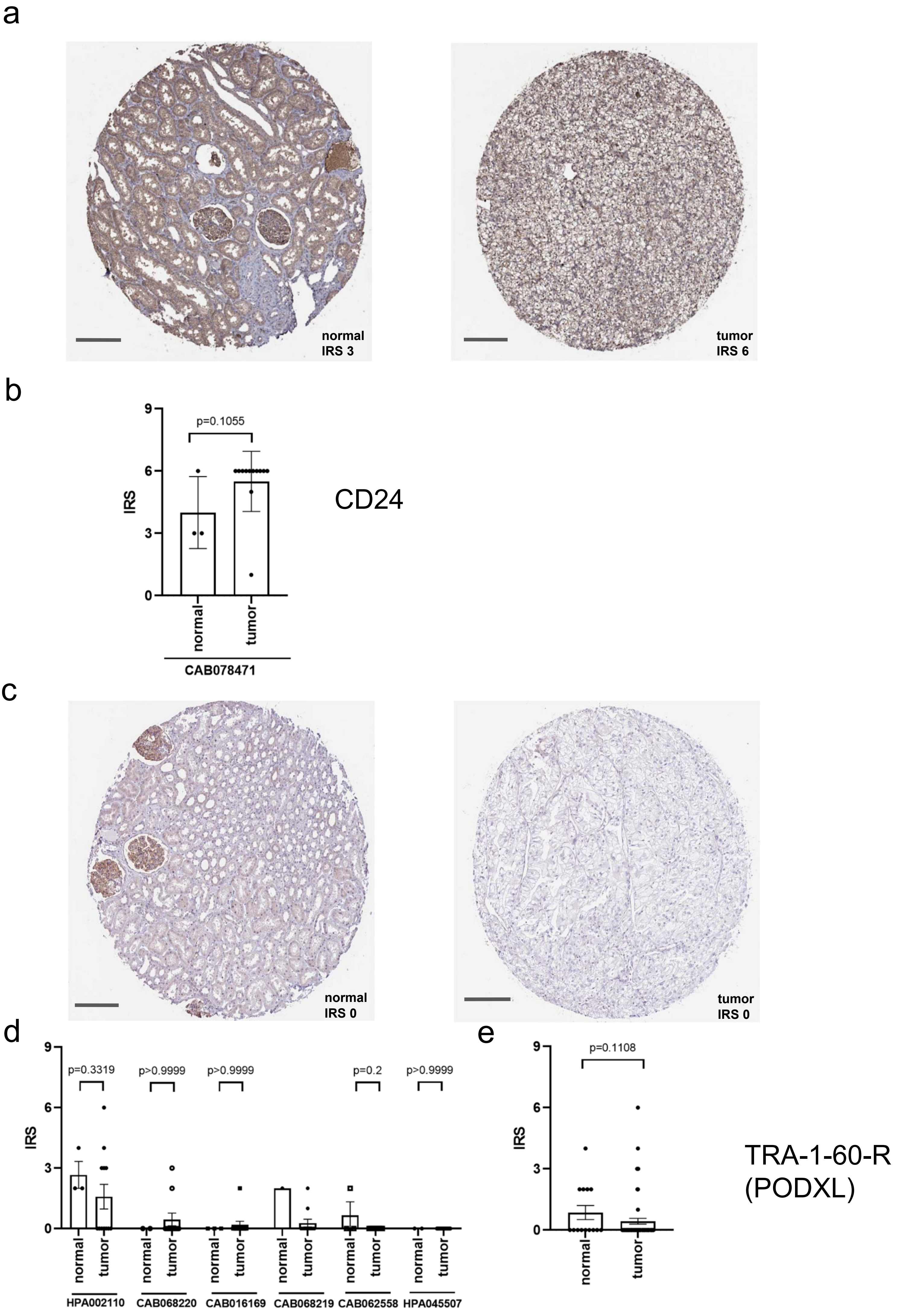

a
normal
IRS 3
tumor
IRS 6
b
p=0.1055
IRS
normal
tumor
CAB078471
CD24
c
normal
IRS 0
tumor
IRS 0
d
p=0.3319
p>0.9999
p>0.9999
p=0.2
p>0.9999
IRS
normal
tumor
HPA002110
CAB068220
CAB016169
CAB068219
CAB062558
HPA045507
e
p=0.1108
IRS
normal
tumor
TRA-1-60-R
(PODXL)

f

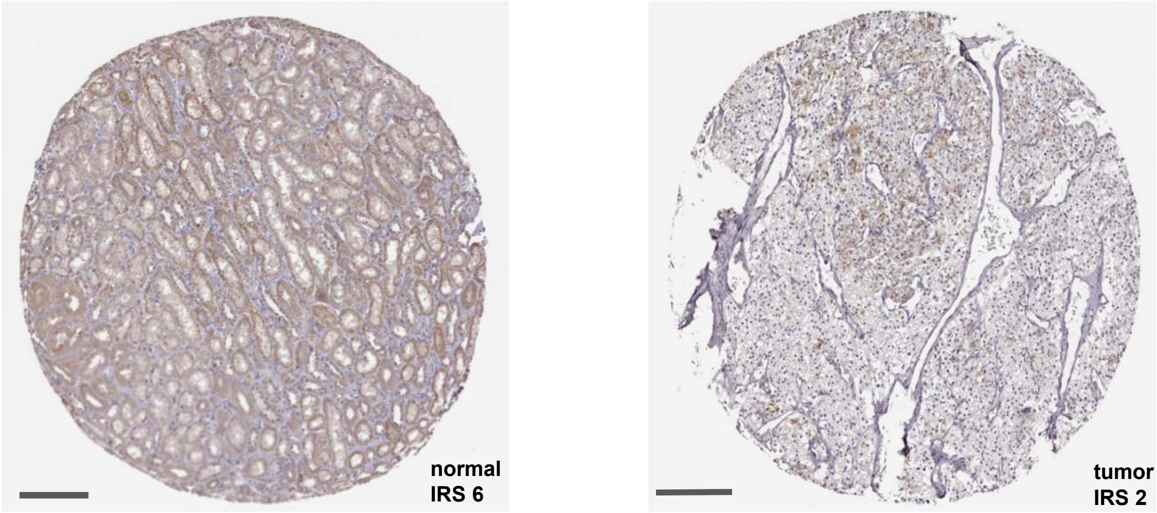

g

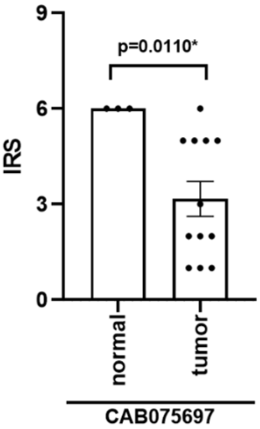

TIM-1

h

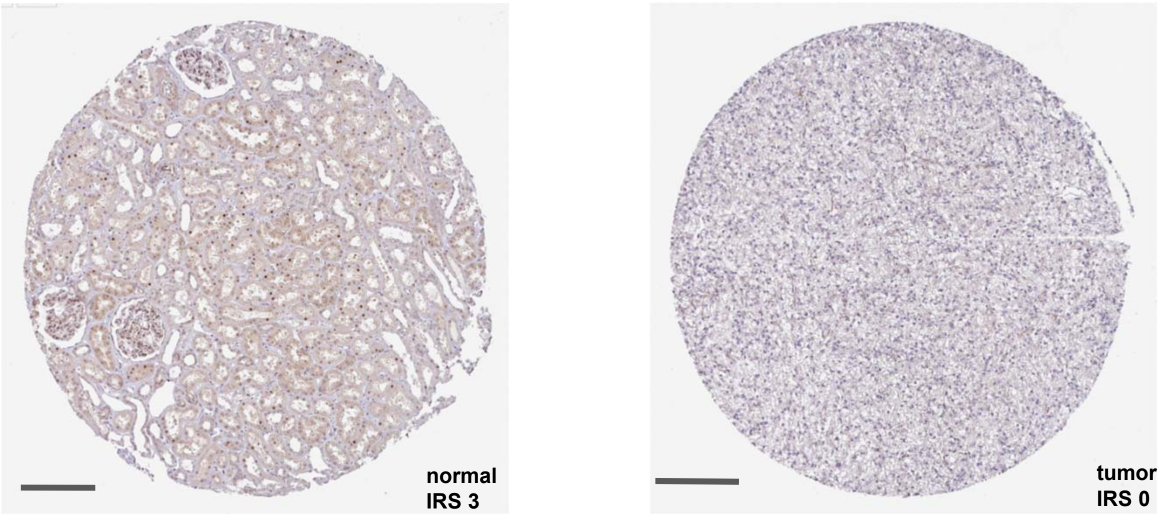

i

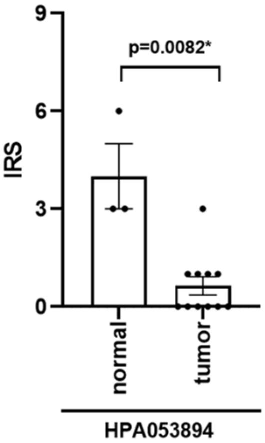

SSEA-4 (TMCC1)

j

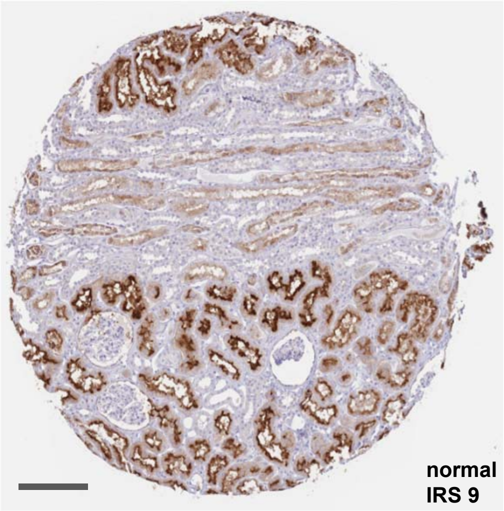

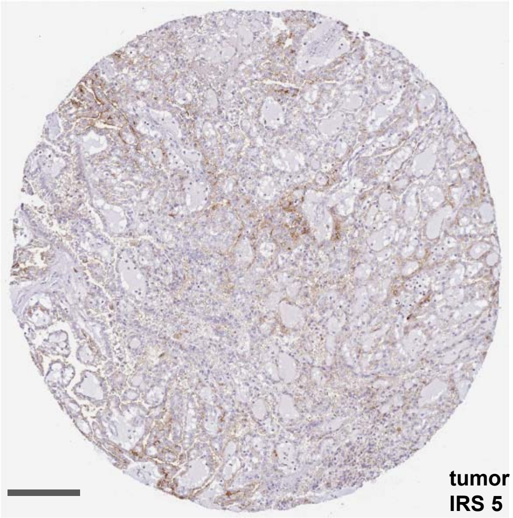

k

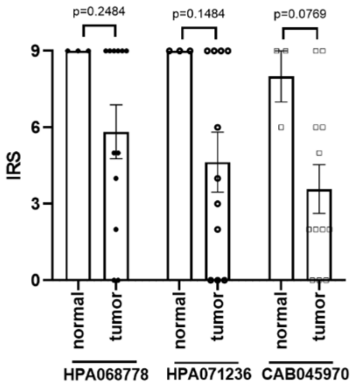

CD26
(DPP4)

l

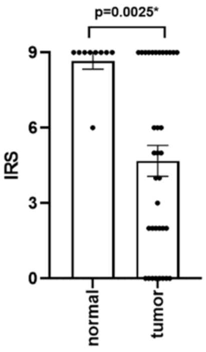

m

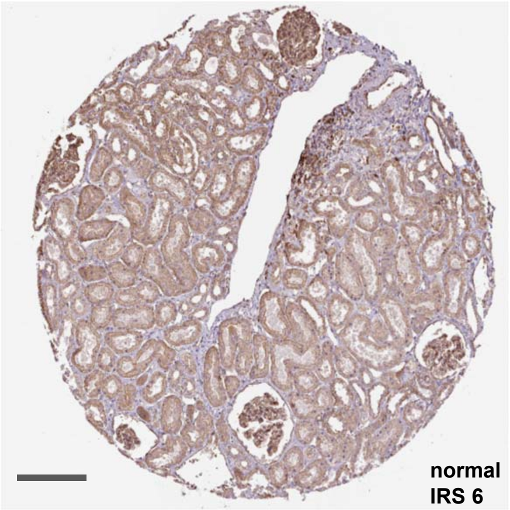

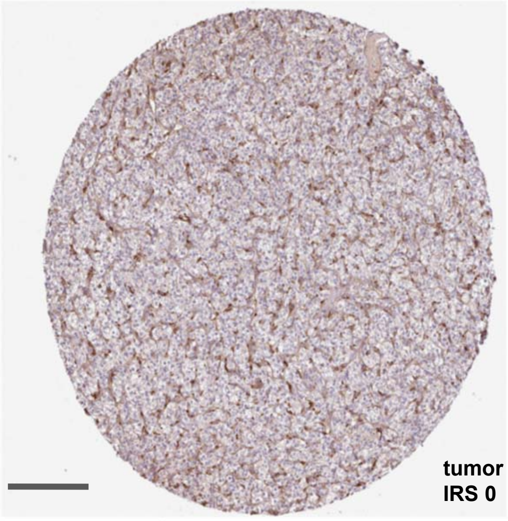

n

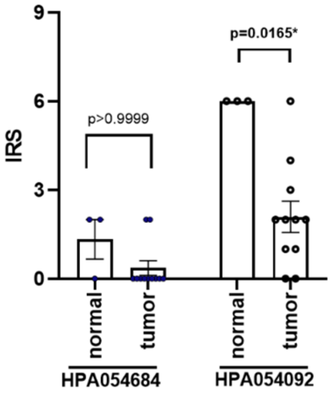

SSEA-3
(B3GALT5)

o

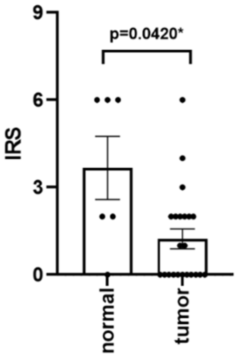

Fig. S7: Expression of CD24, TRA-1-60-R (PODXL), TIM-1, SSEA-4 (TMCC1), CD26 (DPP4) and SSEA-3 (B3GALT5) in healthy vs. tumor tissue based on HPA data. (a) Representative tissue cores from the HPA showing immunohistochemistry (IHC) staining for CD24. Left core, normal kidney; right core, renal cancer. (b) Summarized data for immunoreactive score (IRS) for normal and tumor kidney for a single antibody for CD24. (c) Representative tissue cores from the HPA showing IHC staining for TRA-1-60-R. Left core, normal kidney; right core, renal cancer. (d-e) Summarized data for IRS for normal and tumor kidney from six antibodies shown separately (d) and pooled (e). (f) Representative tissue cores from the HPA showing IHC staining for TIM-1. Left core, normal kidney; right core, renal cancer. (g) Summarized data for IRS for normal and tumor kidney for a single antibody for TIM-1. (h) Representative tissue cores from the HPA showing IHC staining for SSEA-4 (TMCC1). Left core, normal kidney; right core, renal cancer. (i) Summarized data for IRS for normal and tumor kidney for a single antibody for SSEA-4 (TMCC1). (j) Representative tissue cores from the HPA showing IHC staining for CD26 (DPP4). (k-l) Summarized data for IRS for normal and tumor kidney from three antibodies shown separately (k) and pooled (l). (m) Representative tissue cores from the HPA showing IHC staining for SSEA-3 (B3GALT5). Left core, normal kidney; right core, renal cancer. (n-o) Summarized data for IRS for normal and tumor kidney from two antibodies shown separately (n) and pooled (o).
Scale bars, 200 µm
